# Burkholderia pseudomallei infection in a patient with diabetes presenting with multiple splenic abscesses and abscess in the foot: a case report

**DOI:** 10.1186/1757-1626-1-224

**Published:** 2008-10-07

**Authors:** Rahul Dhodapkar, S Sujatha, K Sivasangeetha, G Prasanth, Subhash Chandra Parija

**Affiliations:** 1 Department of Microbiology, Jawaharlal Institute of Postgraduate Medical Education and Research, Pondicherry, PIN 605006, India

## Abstract

**Introduction:**

Melioidosis or infection with *Burkholderia pseudomallei *presents with protean manifestations. We present a case of melioidosis in a diabetic patient from India. The case is presented to highlight the importance of early microbiologic diagnosis and subsequent institution of appropriate therapy to achieve a better prognosis

**Case presentation:**

A male bachelor around 50 years of age from India presented with low grade fever, bilateral ankle swelling and hypochondrial pain. On examination patient had diabetes and had multiple abscesses in bilateral ankle, knee and splenic region. Microbiologic diagnosis revealed the etiologic agent to be *Burkholderia pseudomallei. *Patient was managed with iv ceftazidime and surgical excision.

**Conclusion:**

The case report highlights the importance of early identification of etiologic agent. *B. pseudomallei *identification requires a great deal of clinical suspicion as well as alertness on the part of the medical microbiologist as these isolates are often reported as *Pseudomonas *spp. Correct identification of the etiologic agent is essential as *B pseudomallei *requires prolonged antimicrobial therapy for a better clinical outcome.

## Introduction

Melioidosis is a systemic infection caused by non fermenting gram negative bacilli *Burkholderia pseudomallei*. It is endemic in South East Asia and Australia[1] where it causes infections with protean manifestations. It is now recognized as an emerging infectious disease in India [2]. We present a case report of *B. pseudomallei *infection in a diabetic presenting with multiple splenic abscesses and abscess in foot.

## Case presentation

A male bachelor aged 50 years presented with low grade fever for duration of one month, bilateral ankle swelling and left hypochondrial pain for duration of 15 days with associated symptoms of anorexia, nausea and weight loss. There was no history of travel to any foreign country. He was an agricultural worker by profession.

**Figure 1:**
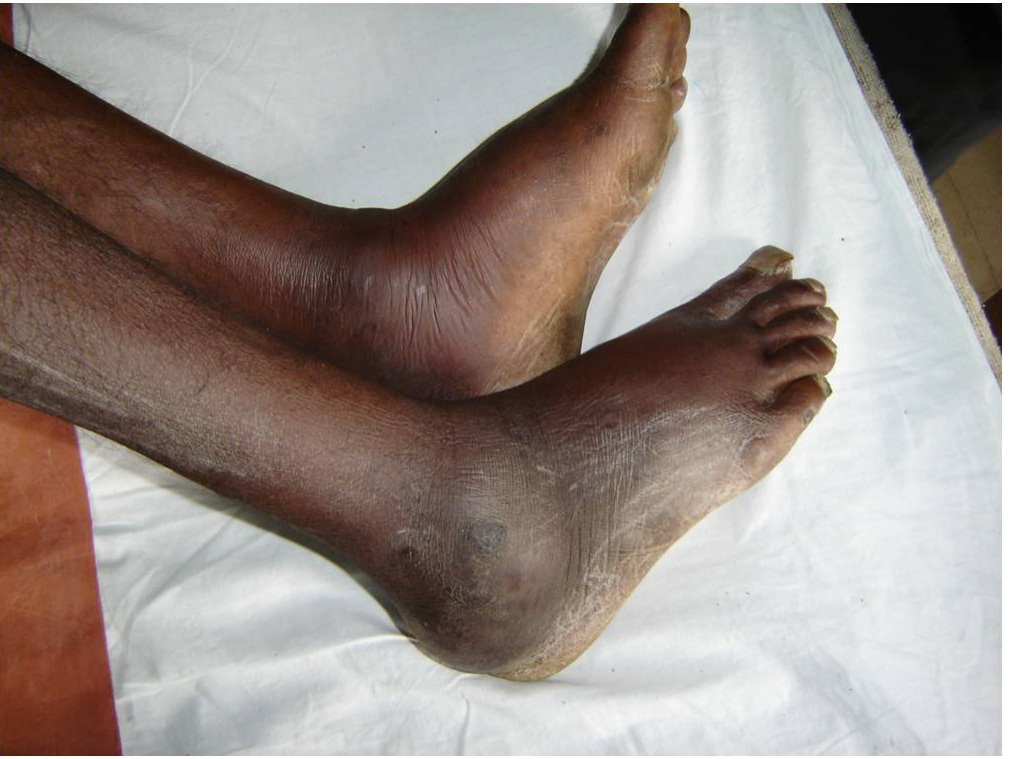
Bilateral ankle swelling.

**Figure 2:**
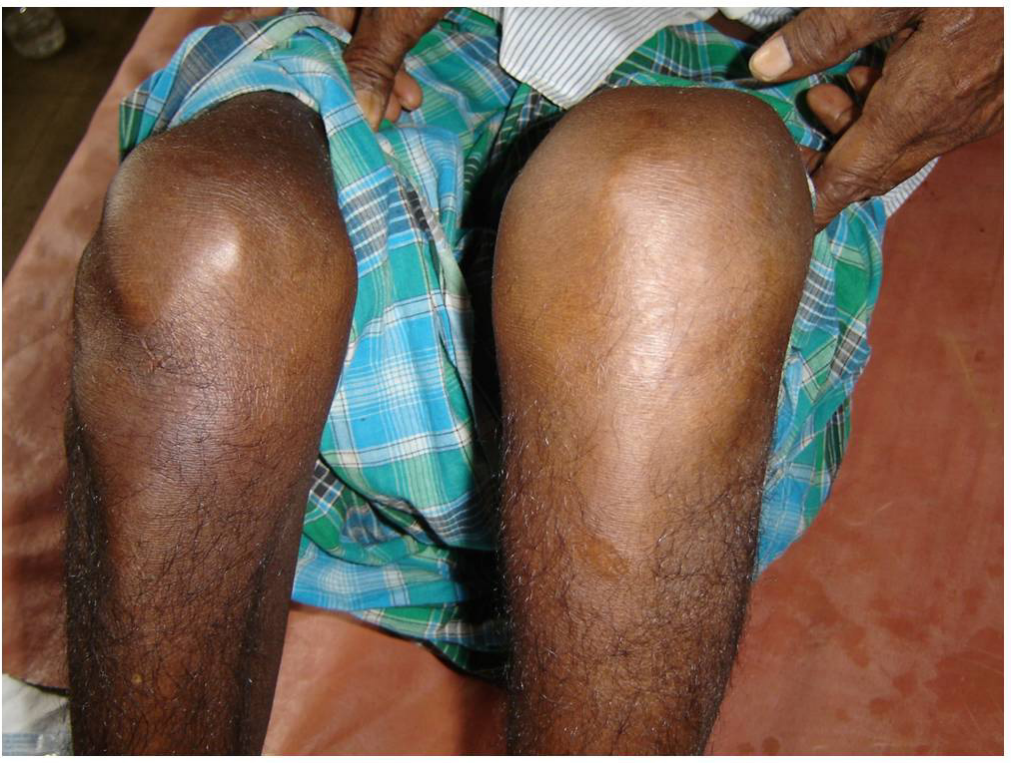
Left knee swelling.

**Figure 3:**
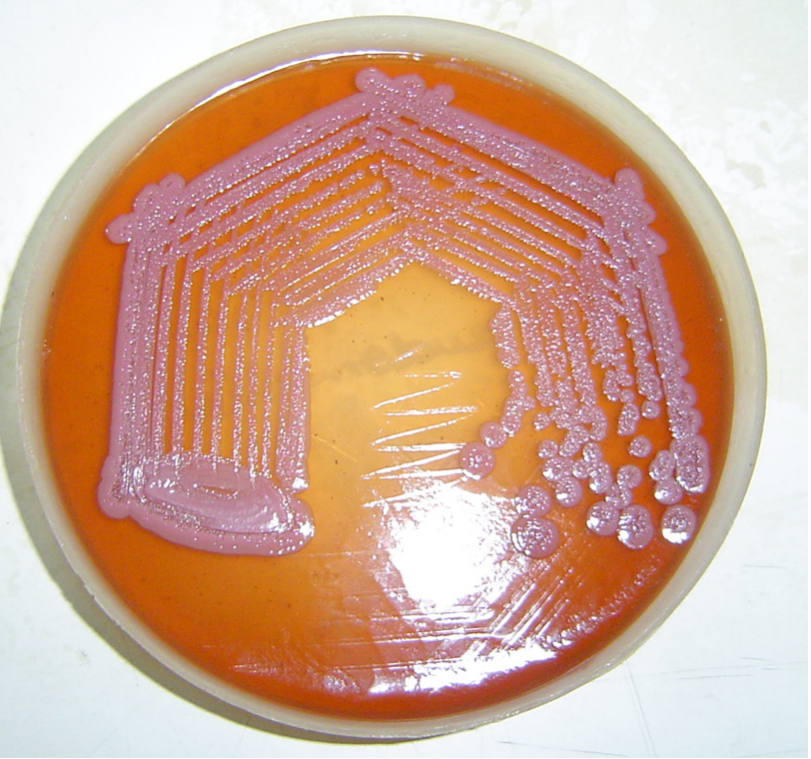
Growth of *Burkholderia pseudomallei *on Mac Conkey agar showing pink, rugose colonies with a metallic sheen.

On examination bilateral swelling in ankle region with tenderness, with restriction in movement of affected joint was observed(Figure 1); per abdomen examination revealed a spleen of 4 cm below the costal margin which was tender on palpation. USG abdomen confirmed the physical finding of an enlarged spleen with multiple hypo echoic lesions, largest being 3 cm in diameter. X ray of ankles was normal with no joint involvement. Ultrasonographic examination revealed a collection of fluid in subcutaneous plane in bilateral ankle region(Figure 2). Investigations revealed neutrophilia, raised ESR and a markedly elevated ALP- 750 U/L. The patient was diagnosed to be a diabetic on the basis of oral glucose tolerance test. Further during the course of his stay in the hospital, the patient developed a swelling in his right knee. At this point a differential diagnosis of tuberculosis, AIDS with multiple site infections, and infective endocarditis were considered. However these were ruled out as specific tests for tuberculosis and HIV were negative, blood culture was negative and there were no cardiac abnormalities detected in the echocardiogram. A diagnostic aspirate from the left ankle revealed frank pus which on gram staining revealed the presence of gram negative bacilli with a typical safety pin appearance. Culture from the pus grew dry wrinkled colonies on blood agar and pinkish rugose colonies on MacConkey's agar(Figure 3). The peculiar appearance on gram staining and colony characters on culture raised the suspicion of *B. pseudomallei*. The isolate was identified as *B. pseudomallei *by standard biochemical methods [3] and was found to be sensitive to ciprofloxacin and ceftazidime. The patient was started on Ceftazidime 2 grams iv given 8 hourly, abscesses were drained surgically, repeat cultures were positive for two weeks but were sterile thereafter; iv antibiotics were continued for one month. Control of the patient's blood sugar was achieved simultaneously and he was discharged after a month. One of the surprising features was that the patient remained afebrile throughout the course of hospitalization.

## Discussion

*Burkholderia pseudomallei *is a soil saprophyte, endemic in south east Asian countries – Vietnam, Thailand and also in Australia[1]. Though inoculation is believed to be the major mode of infection, ingestion and person to person transmission have also been suggested in certain cases. *B. pseudomallei *infections are known for their protean manifestations ranging from systemic manifestations like septicemia, pneumonia & multiple abscesses to asymptomatic infections, local ulcers and abscesses without systemic manifestations. Recently there have been increasing reports of infections with *B. pseudomallei *from India. Cases reported from Indian subcontinent varied from serious manifestations like septicaemia [4,5], septic arthritis[6,7], pneumonia[7] to soft tissue infections like scalp abscess, psoas abscess, gluteal abscess [7]etc.

The patient in question presented with multiple splenic abscesses, abscesses in his feet and went on to develop swelling in his right knee. He was later diagnosed to be a diabetic during his stay in the hospital. Diabetes has been found to be the single most common predisposing factor in a review [8]. No case reports describing sequential swellings in the feet followed by knees were found after a search of existing literature, though progression of cutaneous swellings to necrotizing fascitis has been described [9]. Though the patient had multiple foci of infection he presented with remarkably mild symptoms and there were no complications associated with the management.

## Conclusion

Melioidosis as a differential diagnosis should be an option in multiple abscesses especially in patients with predisposing factors in the Indian subcontinent as there has been an upsurge in case reports of *B. pseudomallei *infections from the region. A very high index of suspicion should be kept both clinically and at laboratory level as cultures of *B. pseudomallei *can very easily be misidentified as those of *Pseudomonas*. Atypical presentations in non endemic areas in patients with predisposing conditions with inconclusive routine investigation should prompt the clinician to ask for a travel history to any of the areas where the organism is endemic or sporadic. The situation can be further complicated by the reactivation of a latent focus of infection acquired many years ago as has been seen in American soldiers returned from the Vietnam War [10,11]. Correct identification of *B. pseudomallei *is essential as treatment of these infections require intensive and prolonged treatment [12,13].

## Abbreviations

USG: Ultrasonography; ESR: Erythrocyte sedimentation rates; ALP: Alkaline phosphatase; AIDS: Acquired immunodeficiency disease; HIV: Human immunodeficiency virus.

## Consent

Written informed consent was obtained from the patient for publication of this case report and accompanying images. A copy of the written consent is available for review by the Editor-in-Chief of this journal.

## Competing interests

The authors declare that they have no competing interests.

## Authors' contributions

RD contributed in preparation of manuscript, SS & KS isolated and characterized the isolate, PG was the physician incharge for the patient, SCP contributed in editing the manuscript. All authors read and approved the final manuscript.
